# Supplementary material for: ProteinShader: illustrative rendering of macromolecules
Source: BMC Struct Biol. 2009 Mar 30;9:19. doi: 10.1186/1472-6807-9-19 (PMC2672931; doi:10.1186/1472-6807-9-19)
Supplement: Additional file 1 — ProteinShader program without source code. This compressed file contains the complete ProteinShader program including associated libraries, but no source code. A README.txt file gives an overview of the ProteinShader distribution, and the index.html file in the help subdirectory has directions on getting started with the program as well as a set of tutorials. [file 1472-6807-9-19-S1.zip › ProteinShader-beta-0_9_4-binary/help/api/org/proteinshader/structure/exceptions/class-use/InvalidIDException.html]

Uses of Class org.proteinshader.structure.exceptions.InvalidIDException (ProteinShader API)


|  |  |  |
| --- | --- | --- |
| **Overview** \| **Package** \| **Class** \| **Use** \| **Tree** \| **Deprecated** \| **Index** \| **Help** | |  |
| PREV   NEXT | **FRAMES**    **NO FRAMES**     **All Classes** |  |


---


## **Uses of Class org.proteinshader.structure.exceptions.InvalidIDException**

| Packages that use InvalidIDException | |
| --- | --- |
| **org.proteinshader.structure** | Holds the classes that store information from a Protein Data Bank file: Structure, Model, Chain, AminoAcid, Heterogen, Water, Atom, Bond, Helix, BetaStrand, Loop, *etc*. |

Uses of InvalidIDException in org.proteinshader.structure

| Methods in org.proteinshader.structure that throw InvalidIDException | |
| --- | --- |
| `AminoAcid` | `Chain.addNewAminoAcid(AminoAcidEnum type, String residueID)`             Creates a new AminoAcid with the type and Residue ID given as arguments, adds the AminoAcid to the Chain's collection of AminoAcids, and returns a reference to the new AminoAcid. |
| `Atom` | `AminoAcid.addNewAtom(int serialNo, AtomEnum type, String atomID, double temperature, int charge, double occupancy, String altLocation, double x, double y, double z, VisibilityEnum visibility)`             Creates a new Atom with the type and Atom ID given as arguments, adds the Atom to the Residue's collection of Atoms, and returns a reference to the new Atom. |
| `Atom` | `Residue.addNewAtom(int serialNo, AtomEnum type, String atomID, double temperature, int charge, double occupancy, String altLocation, double x, double y, double z, VisibilityEnum visibility)`             Creates a new Atom with the type and Atom ID given as arguments, adds the Atom to the Residue's collection of Atoms, and returns a reference to the new Atom. |
| `Atom` | `Structure.addNewAtom(int atomSerialNo, AtomEnum atomType, String atomID, String aminoAcidType, String residueID, String chainID, String modelID, double temperature, int charge, double occupancy, String altLocation, double x, double y, double z, VisibilityEnum visibility)`             Creates a new atom with the atomID given as an argument, adds the new Atom to the requested Model-Chain-AminoAcid, and then returns a reference to the new Atom. |
| `BetaStrand` | `Chain.addNewBetaStrand(String betaStrandID, String betaStrandNumber, String sheetID, String startResidueID, String endResidueID, int sense, int strandsInSheet)`             Creates a new BetaStrand with the BetaStrand ID given as an argument, adds the BetaStrand to the Chain's collection of BetaStrands, and returns a reference to the new BetaStrand. |
| `void` | `Structure.addNewBetaStrand(String chainID, String betaStrandID, String betaStrandNumber, String sheetID, String startResidueID, String endResidueID, int sense, int strandsInSheet)`             The BetaStrand will be added to the equivalent Chain on each Model, but this addition will only work if the sequence of Residues that the BetaStrand refers to have already been added. |
| `void` | `Model.addNewBetaStrand(String chainID, String betaStrandID, String betaStrandNumber, String sheetID, String startResidueID, String endResidueID, int sense, int strandsInSheet)`             The BetaStrand will be added to the Chain only if the sequence of Residues that the BetaStrand refers to has already been added. |
| `Category` | `Description.addNewCategory(String categoryName)`             Creates a Category, adds it to a hash, and then returns the new Category. |
| `Chain` | `Model.addNewChain(String chainID)`             Creates a new Chain with the chainID given as an argument, adds the Chain to the Model's collection of Chains, and returns a reference to the new Chain. |
| `Helix` | `Chain.addNewHelix(String helixID, String serialNo, String startResidueID, String endResidueID, HelixEnum type)`             Creates a new Helix with the Helix ID given as an argument, adds the Helix to the Chain's collection of Helices, and returns a reference to the new Helix. |
| `void` | `Structure.addNewHelix(String chainID, String helixID, String serialNo, String startResidueID, String endResidueID, HelixEnum type)`             The Helix will be added to the equivalent Chain on each Model, but this addition will only work if the sequence of Residues that the Helix refers to have already been added. |
| `void` | `Model.addNewHelix(String chainID, String helixID, String serialNo, String startResidueID, String endResidueID, HelixEnum type)`             The Helix will be added to the Chain only if the sequence of Residues that the Helix refers to has already been added. |
| `Atom` | `Structure.addNewHetAtom(int atomSerialNo, AtomEnum atomType, String atomID, String hetName, String residueID, String chainID, String modelID, double temperature, int charge, double occupancy, String altLocation, double x, double y, double z, VisibilityEnum visibility)`             Creates a new atom with the atomID given as an argument, adds the new Atom to the requested Model-Chain-(Heterogen or Water), and then returns a reference to the new Atom. |
| `Heterogen` | `Chain.addNewHeterogen(String name, String residueID)`             Creates a new Heterogen with the name and Residue ID given as arguments, adds the Heterogen to the Chain's collection of Heterogens, and returns a reference to the new Heterogen. |
| `void` | `Description.addNewLine(String categoryName, String recordName, String line)`             Adds a line to the requested Category/Record. |
| `Loop` | `Chain.addNewLoop(String loopID, String startResidueID, String endResidueID)`             Creates a new Loop with the Loop ID given as an argument, adds the Loop to the Chain's collection of Loops, and returns a reference to the new Loop. |
| `Model` | `Structure.addNewModel(String modelID)`             Creates a Model, adds it to the Structure, and returns a reference to the new Model. |
| `Record` | `Category.addNewRecord(String recordName)`             Creates a Record, adds it to a hash, and returns the new Record. |
| `Water` | `Chain.addNewWater(String residueID)`             Creates a new Water with the Residue ID given as an argument, adds the Water to the Chain's collection of Waters, and returns a reference to the new Water. |
| `String` | `Structure.processID(String id, String typeOfID)`             Returns the ID after trimming any leading or trailing whitespace. |
| `String` | `IDTest.processID(String id, String typeOfID)`             Returns the ID after trimming any leading or trailing whitespace. |
| `String` | `Residue.processID(String id, String typeOfID)`             Returns the ID after trimming any leading or trailing whitespace. |
| `String` | `Chain.processID(String id, String typeOfID)`             Returns the ID after trimming any leading or trailing whitespace. |
| `String` | `Model.processID(String id, String typeOfID)`             Returns the Model ID after trimming any leading or trailing whitespace. |
| `String` | `Atom.processID(String id, String typeOfID)`             Returns the ID after trimming any leading or trailing whitespace. |
| `String` | `Region.processID(String id, String typeOfID)`             Returns the ID after trimming any leading or trailing whitespace. |
| `String` | `Heterogen.processID(String id, String typeOfID)`             Overrides the processID() method of Residue in order to add guarantee that the residueID of a Heterogen always begins with the HETEROGEN\_PREFIX. |

Constructors in org.proteinshader.structure that throw InvalidIDException

`AminoAcid(AminoAcidEnum type,
String residueID,
String chainID,
String modelID,
String structureID)`
  
          Constructs an AminoAcid with the type and IDs given as arguments.

`Atom(int serialNo,
AtomEnum type,
String atomID,
String residueID,
String chainID,
String modelID,
String structureID,
double temperature,
int charge,
double occupancy,
String altLocation,
double x,
double y,
double z,
VisibilityEnum visibility)`
  
          This constructor uses the type argument (an AtomEnum) to set the
default radius and CPK color for the Atom.

`BetaStrand(String betaStrandID,
String betaStrandNumber,
String sheetID,
String startResidueID,
String endResidueID,
Chain chain,
int sense,
int strandsInSheet)`
  
          Constructs a BetaStrand.

`Category(String name)`
  
          Constructs a Category with the name specified as an argument.

`Chain(String chainID,
String modelID,
String structureID)`
  
          Constructs a Chain with the requested chainID, modelID, and
structureID.

`Helix(String helixID,
String serialNo,
String startResidueID,
String endResidueID,
Chain chain,
HelixEnum type,
HelixShapeEnum shape)`
  
          Constructs a Helix.

`Heterogen(String name,
String residueID,
String chainID,
String modelID,
String structureID)`
  
          Creates a Heterogen with the name and IDs given as arguments
(after adding the HETEROGEN\_PREFIX ("HET\_") to the beginning of
residueID).

`Loop(String loopID,
String startResidueID,
String endResidueID,
Chain chain)`
  
          Constructs a Loop.

`Model(String modelID,
String structureID)`
  
          Constructs a Model with the requested modelID and structureID.

`Record(String categoryName,
String recordName)`
  
          Constructs a Record with the name specified as an argument.

`Region(String startResidueID,
String endResidueID,
Chain chain)`
  
          Constructor for use by subclasses.

`Residue(String residueID,
String chainID,
String modelID,
String structureID)`
  
          Constructor for use by subclasses.

`Structure(String structureID)`
  
          Constructs a Structure with the requested structureID.

`Water(String residueID,
String chainID,
String modelID,
String structureID)`
  
          Constructs a Water with the requested residueID.

---


|  |  |  |
| --- | --- | --- |
| **Overview** \| **Package** \| **Class** \| **Use** \| **Tree** \| **Deprecated** \| **Index** \| **Help** | |  |
| PREV   NEXT | **FRAMES**    **NO FRAMES**     **All Classes** |  |


---

# *Copyright © 2007-2008*
